# A comparison between different models of delivering maternal cash transfers in Myanmar

**DOI:** 10.1093/heapol/czae048

**Published:** 2024-06-27

**Authors:** Elisa M Maffioli, Nicholus Tint Zaw, Erica Field

**Affiliations:** Department of Health Management and Policy, University of Michigan School of Public Health, 1415 Washington Heights, Ann Arbor, MI 48109, United States; International Food Policy Research Institute, 1201 Eye St., NW, Washington, DC 20005, United States; Department of Economics, Duke University, 419 Chapel Drive, Durham, NC 27710, United States

**Keywords:** Health workers, nutrition, maternal and child health, health policy, developing countries

## Abstract

As part of a randomized controlled trial conducted in Myanmar between 2016 and 2019, we explore the performance of a maternal cash transfer program across villages assigned to different models of delivery (by government health workers vs loan agents of a non-governmental organization) and identify key factors of success. Measures include enrolment inclusion and exclusion errors, failures in payment delivery to enrolled beneficiaries (whether beneficiaries received any transfer, fraction of benefits received and whether there were delays and underpayment of benefit amounts) and whether beneficiaries remained in the program beyond eligibility. We find that women in villages where government health workers delivered cash transfers received on average two additional monthly transfers, were 19.7% more likely to receive payments on time and in-full and were 14.6% less likely to stay in the program beyond eligibility. With respect to the primary health objective of the program—child nutrition—we find that children whose mother received cash by government health workers were less likely to be chronically malnourished compared to those whose mother received cash by loan agents. Overall, the delivery of cash transfers to mothers of young children by government health workers outperforms the delivery by loan agents in rural Myanmar. Qualitative evidence suggests two key factors of success: (1) trusted presence and past interactions with targeted beneficiaries and complementarities between government health workers’ expertise and the program; and (2) performance incentives based on specific health objectives along with top-down monitoring. We cannot exclude that other incentives or intrinsic motivation also played a role.

Key messages

- Social welfare programs are the cornerstone of policy for improving living conditions.
- We compare different models of delivering cash transfers.
- We find that government health workers outperformed loan agents of a non-governmental organization.
- Focus Group Discussions suggest that complementarities in the incentives of health workers in providing cash and health services

## Introduction

Social welfare programs are the cornerstone of government policy for improving the living conditions of their citizens (Shahidi *et al*., 2019). However, public sector resource constraints frequently limit the ability of governments in poor settings to deliver wide-reaching social programs, generating a reliance on non-state actors for program delivery, particularly in the health care sector. Governments around the world face severe human resources and financial challenges in providing health services (Kirigia *et al*., 2004; Kabene *et al*., 2006; Masiye, 2007), and non-state actors, either from local or international non-governmental organizations (NGOs) or the private sector increasingly fill the gap in public sector service delivery (Springman, 2022).

It is an open question as to whether this shift has implications for the quality of health services, and which are the key factors determining success in public goods delivery. While there are several arguments for why governments have a natural advantage in public goods provision, when state capacity is weak, non-state actors may be more effective in managing public goods delivery.^1^ A recent review of the literature (Rao, 2015) found no conclusive evidence that any model of management (i.e. public, private or mixed) was more efficient, though a Bayesian meta-analysis evaluating 20 types of international development interventions, found that government-implemented programs had smaller effect sizes than NGO-implemented ones (Vivalt, 2020).

The answer depends on whether the advantages of relying on government employees outweigh the potential limitations of doing so. There are several ways in which government delivery is likely to diverge from non-governmental delivery of public services. First, government employees may face a different level of *trust* by community members than do non-governmental employees, which could limit the effectiveness of program delivery. In settings with weak public institutions, trust in government employees may be low, although in other settings, NGOs may be viewed as outsiders even if they are domestic. Key factors will be the overall level of trust in government and the degree of social distance between providers and community members, which depends on the local presence of providers and their past interaction with targeted beneficiaries. Second, performance could diverge because of differences in worker or organizational expertise, which will again depend on the degree to which the NGO has experience working in the community and/or service-specific expertise. Related to our setting, a study in Bangladesh (Khan and Ahmed, 2002) found that delivery of nutrition services was more cost-effective when run through government nutrition centres rather that non-government ones.^2^

Third, non-governmental providers may differ from government providers in service delivery effectiveness on account of differences in ‘performance incentives’ across state and non-state actors. For example, Banerjee *et al*. (2008) showed that government supervisors in Indian public clinics sabotaged an NGO program that monitored absenteeism by nurses. While initially the monitoring system on government nurses was effective, over time there was collusion between the local health administration and the nurses, making the program ineffective after only 18 months. Relatedly, the quality of program delivery may depend on the ‘(intrinsic or extrinsic) motivation’ of the actors involved. Ghatak (2005) predicts that NGOs perform better than government organizations even when the programs have equivalent resources because NGO employees are driven by moral, ideological and altruistic motives rather than financial ones (Ghatak, 2005; Werker and Ahmed, 2008). On the other hand, because NGOs are often funded by donors, their incentives may be shaped by donor preferences more than industry best practices, which may create inefficiencies (Werker and Ahmed, 2008). For example, Reinikka and Svensson (2010) found that religious not-for-profit health care providers provided higher quality care than government facilities because of their intrinsic motivation to serve poor people.

Given that cash transfer programs now reach over one billion people globally and are one of the preferred social welfare strategies employed by governments worldwide (Bastagli *et al*., 2016), it is critical to understand key success factors for delivering these types of programs. In this study, we compare the delivery performance of a maternal cash transfer program implemented through government health workers vs loan agents of an international NGO, in rural Myanmar. In 2014, the Government of Myanmar, supported by the Department of Social Welfare at the Ministry of Social Welfare, Relief and Resettlement, launched its National Social Protection Strategy. One of the flagship programs of the strategy was a universal cash transfer program for pregnant women and mothers of children <2 years, which was implemented in the Pakokku township, in the Dry Zone of Myanmar between 2016 and 2019.^3^ As Myanmar has one of the highest rates of stunting in the Asia Pacific region (35%), the program was born out of recognition that the first 1000 days of life comprise a critical period for physical and cognitive development later in life (Almond and Currie, 2011; Doyle, 2019). Thus, improving the nourishment of mothers and young children has the potential to positively impact development and productivity over the life course, and, consequently, increase economic growth through future generations of healthier individuals (Engle *et al*., 2007; Hoddinott *et al*., 2013; Richter *et al*., 2017).

This program also presented a unique opportunity to test the relative performance of state vs non-state program delivery and identify key success factors because two different modes of delivery were implemented in different parts of the study setting. A total of 44 villages (22 GOV, 22 NGO) were pair-matched by propensity score based on pre-existing socio-demographic characteristics, and outcome data were collected from more than 9000 study participants. Our empirical analysis investigates the relative performance of state and non-state actors and the implications for beneficiary outcomes. Through focus group discussions with government health workers, we explore the reasons behind observed differences in performance. In doing so, we shed light on the benefits of implementing a maternal cash transfer program through an existing infrastructure of government health workers rather than agents of an NGO in an under-resourced setting.

## Methods

This study is part of a larger randomized controlled trial that investigates the impacts of a maternal cash transfer program, combined with social behavioural change communication (SBCC), on child nutrition (Field and Maffioli, 2024).^4^ The experiment was implemented in three townships of the Dry Zone of Myanmar. Triplets of villages, grouped together by a mapping algorithm based on geographic proximity alone, that were located within a 2-h radius of the main city and served by sub-rural health centres (clusters) were randomized into two intervention arms [(1) Cash-only and (2) Cash + SBCC] and a control group.

Women were eligible to receive benefits starting in the 4th month of pregnancy and continuing until their child reached 24 months of age. Each beneficiary in a treated village received a monthly cash transfer of 10 000 MMK (∼$4.75) meant to assist with the purchase of nutritious foods and access to antenatal and postnatal care. The program is based on a ‘soft’ conditionality: mothers were encouraged to attend nutrition and hygiene promotion sessions, were referred to clinics to attend ante- and postnatal services and reminded of their children’s immunization according to the national schedule. However, if they did not take any action, they were not excluded from the cash transfer program, as there was no tracking of health care usage by mothers.

### Sample and interventions

Within the three townships, we identified 120 sub-rural health centre catchment areas within 2 h from the main town and feasible for program implementation. Natural clusters were then created based on closer proximity through *K*mean algorithm, and within each natural cluster, triplets of health catchment areas were randomly matched by closest distance through the unique permutations’ efficient algorithm. Using this method, a total of 102 catchment areas were matched into 34 triplets and selected for the main randomized controlled trial as they happen to be physically closer, while the remaining 18 catchment areas were not found to have a match within the clusters and were excluded from the main study. We use all villages remaining in one of the three townships (Pakokku), which were not selected to be part of the main randomized controlled trial, for this secondary study. Specifically, among these remaining 18 (unmatched) catchment areas, 4—encompassing 22 villages—were located in Pakokku township. Under the direction of the Department of Social Welfare, all 22 villages in Pakokku were selected for government-run program management, in which government health workers delivered the cash transfers. This provided a unique opportunity to compare a maternal cash transfer program in which delivery was managed by loan agents of an NGO with the exact same cash transfer program running simultaneously in neighbouring villages in which delivery was managed by government health workers.

Specifically, the two interventions entailed the following:

Government health workers (GOV): Government health workers, with the support of Village Health Committees—composed of local leaders and health workers—enrolled beneficiaries and delivered cash transfers. The 11 government health workers in our study were all female. As reported in the qualitative data collection, they were all from villages within Pakokku township, and mostly lived in the sub-rural health centre in their current assigned village. They covered between 3 and 12 villages and a population of 2000–8000 beneficiaries. All government health workers (independent of coverage area) received ∼$150 per month, including a stipend, support for uniform and food and an additional hardship allowance ($38) to reach remote locations by the Ministry of Health and Sports. Most reported having a midwifery diploma, which in Myanmar is obtained with 2 years of midwifery school managed by the Ministry of Health and Sports. Before the maternal cash transfer program was launched, government health workers were already embedded in the communities and served as health providers for pregnant women and their young children. Hence, mothers may have already interacted with them on a regular basis during pregnancy and birth, and for seeking health care for young children. This model was implemented in 22 villages in Pakkoku located in the 18 sub-rural health catchment areas that remained unmatched after an algorithm matched 102 sub-rural health centre catchment areas into geographic triplets as part of the main randomized controlled trial (Field and Maffioli, 2024).

Loan agents of an NGO (NGO): a non-profit international development organization that delivers microfinance services, called Pact Global Microfinance, delivered cash transfers to beneficiaries. Government health workers, with the support of Village Health Committees, were still responsible for enrolling beneficiaries and validating enrolment, but Pact Global Microfinance managed cash disbursements through its network of mostly female loan agents. While the organization had an office in the main town in Pakokku township, the loan agents travelled to villages for their microfinance activities as well as to disburse cash for this program. Each loan agent was responsible for 8–10 villages, and used existing women’s group activities where women congregated, such as women’s saving group meetings, to deliver the monthly cash transfers. Typically, loan agents were not local to the villages and would come into the community for their business. This model was implemented in 48 villages in Pakkoku that were assigned to the ‘Cash Only’ treatment arm of the main randomized controlled trial.

While the assignment of villages to the government-led model vs the NGO-managed model was not randomized, the main reasons that villages remained unmatched to triplets and hence were assigned to the government-led model was arguably quasi-random since it occurred when clusters selected by geographic proximity contained an uneven number of catchment areas (not a multiple of 3). That is, clusters that contained 20 catchment areas were matched into six triplets, necessarily leaving two unmatched catchment areas within that cluster. However, within clusters, it was more statistically likely that remote catchment areas went unmatched and ended up in the government-led group.

Thus, to improve on the comparability between villages receiving the government vs the NGO model, we applied a propensity score matching algorithm and matched villages (one to one, no replacement) based on socio-demographic characteristics collected prior to the intervention during a village-level census (February 2016). Table 1 (and Supplementary Appendix Table S1) presents the list of variables used in the matching algorithm, and Supplementary Appendix Figure S1 shows the bias reduction across the covariates after implementing the propensity score matching. This approach allows us to construct comparison groups that are balanced on observable characteristics. Our resulting analysis sample includes all 22 villages assigned to the government model and another 22 NGO-led villages that were deemed the closest match on observable characteristics. An alternative specification that considers all 70 government-led (22) and NGO-led (48) cash transfer villages in Pakokku township is also presented as a robustness check. The empirical analysis also adjusts the standard errors for the small number of clusters in this secondary analysis (14 or 13 in the full or propensity score matched sample, respectively).

**Table 1.** Village characteristis

	Mean (GOV)	Mean (NGO)	Diff.
Number of households in the village	137.5	169.5	−32.05 (33.72)
Prop. of literate population in the village	0.757	0.782	−0.0250 (0.0514)
Prop. of villages with agriculture as main livelihood	0.864	0.909	−0.0455 (0.0977)
Prop. of villages with livestock as main livelihood	0.364	0.455	−0.0909 (0.151)
Prop. of villages with casual labour as main livelihood	0.909	0.909	0 (0.0887)
Prop. of villages in dry-land farming low land	0.455	0.455	0 (0.154)
Prop. of villages reachable by car in all weather	0.727	0.591	0.136 (0.145)
Prop. of villages that have electricity (grid)	0.227	0.136	0.0909 (0.118)
Prop. of villages with primary school	0.545	0.591	−0.0455 (0.153)
Prop. of villages with local market	0.909	0.955	−0.0455 (0.0775)
Average distance to large market (km)	34.45	36.59	−2.136 (6.360)
Average distance to small market (km)	24	29	−5 (4.800)
Prop. of villages with a health facility	0.182	0.182	0 (0.119)
Prop. of villages that have a midwife	0.227	0.273	−0.0455 (0.133)
Prop. of villages without any water shortage	0.364	0.364	0 (0.148)

Notes: The table presents the balance check of village characteristics for government (GOV) and NGO cash delivery model. Data are from the 2016 census data collection. ***P <0.01, **P <0.05, *P <0.1. The sample includes all villages: 22 from GOV and 22 from NGO.

### Empirical analysis

Our analysis of program performance relies on four sources of data. First, we use endline data collected shortly after program completion, which was ∼30 months after the start of the program for all beneficiaries (between October 2018 and March 2019). The endline sample includes all 643 pregnant women who were initially enrolled in the study villages, along with an additional sample of 615 women of fertile age (15–45 years old) not identified as pregnant at the time of enrolment who were randomly sampled from a listing of villagers. These 1258 women in the endline sample gave birth to a total of 943 children who were enrolled in the program over the course of the study. Along with health and economic outcomes, our endline survey collected self-reported data on program experience as well as child anthropometrics measured by trained enumerators. Second, in order to explore leakages in program delivery, we also conducted monitoring activities in which we interviewed all women >14 years of age in study villages in the midst of program delivery, giving us a dataset on benefits receipt and experiences with the ongoing program from a total of 8315 women. Third, we use village-level census data collected in February 2016 before any intervention, to conduct the propensity score matching exercise. Finally, we conducted post-program qualitative interviews with government health workers to gather information on their experiences with program administration (see details in Field and Maffioli, 2024).

The main outcomes of interest relate to program performance and include the following: the provision of cash transfers to ineligible individuals (inclusion errors), the non-provision to eligible individuals (exclusion errors), whether the beneficiary received any transfer, the total number of transfers received, whether the beneficiary received the correct frequency and cash amount of transfers, and whether beneficiaries remained in the program beyond eligibility (after their child turned 2 years old).^5^ We also explore how the management model corresponds to nutrition goals of the program by looking at recipient outcomes, including child stunting, mothers’ knowledge and behaviour related to nutrition practices and cash usage. Specifically, we use child height and age measurements to construct height for age *z*-scores (HAZ), a well-validated anthropometric measure of chronic malnutrition, using the World Health Organization’s (WHO) child growth standards (World Health Organization, 2006). A HAZ value of −1 indicates that, given sex and age, a child’s height is one standard deviation below the median child in her age/sex reference group. We also construct an indicator of stunting that equals one if HAZ < −2; an indicator of severe stunting that equals one if HAZ < −3; and an indicator of moderate stunting that equals one if −3 ≤ HAZ < −2. Note that the number of observations in the analysis changes depending on the level of analysis (mother-level or child-level) and the construction of the outcome, which can pertain to all women interviewed, to only eligible women, to only ineligible women or to only women enrolled in the program.

The empirical specification follows an ordinary least squares model:


(1) $${{\mathrm{Y}}_{{\mathrm{iv}}}}{ = \alpha + \beta *GO}{{\mathrm{V}}_{\mathrm{v}}}{\mathrm{ + y*}}{{\mathrm{X}}_{{\mathrm{iv}}}}{\mathrm{ + }}{{\varepsilon }_{\mathrm{y}}}$$

where *i* refers to an individual in the sample (woman or child) in village *v. Y_iv_* is the outcome for individual *i* in village *v; GOV_v_* is an indicator taking value 1 if the cash transfers were delivered through government health workers (GOV), and 0 if the cash transfers were delivered through Pact Global Microfinance loan agents (NGO). *X_iv_* is a vector of individual and village controls including woman’s age and education, and head of household’s age and education for the woman-level analysis; and child’s sex and age, mother’s age and education, and head of household’s age and education for the child-level analysis; distance to large and small markets, main source of livelihood (agriculture, livestock or casual labour), availability of government-provided electricity and participation in a concurrent water, sanitation and hygiene (WASH) intervention. Standard errors *ε_v_* are clustered at the health centre catchment area level, the unit of propensity score matching and treatment assignment in the main randomized controlled trial. Standard errors are calculated using wild bootstrapping to address potential concern over the small number of clusters. This type of inference is typically used to account for within-group dependence in estimating standard errors in a regression framework when there are 5–30 clusters (Cameron *et al*., 2008). Assuming that standard errors are uncorrelated with management model in the matched sample, *β* can be interpreted as the causal effect of the government model on the outcomes of interest.

## Results

### Village characteristics

Table 1 describes the socio-demographic characteristics of matched villages in the government (22) and NGO (22) interventions. In the analysis sample, villages have about 150 households, >70% of the population is literate, and the main source of livelihood is either casual labour or agriculture. A small percentage of villages have electricity and only half of them have a primary school. Eighteen per cent of villages have a health facility, >20% are served by a midwife, and almost all of them have access to a local market.

Importantly, we find that there are no statistically significant differences across villages according to the type of delivery model, consistent with the fact that propensity score matching creates comparison groups that are balanced on observable characteristics. More surprisingly, Supplementary Appendix Table S1 also shows similar results for the full (unmatched) sample of program villages in Pakkoku (22 government and 48 NGO villages): villages assigned to the government model look similar to villages assigned to the NGO model, with the exception of electricity and distance to the closest small market. This suggests that villages within sub-rural health catchment areas that were unmatched into geographically close triplets through the Kmean algorithm are largely similar to those that were matched and selected for the main randomized controlled trial. Thus, those villages in sub-rural catchment areas excluded from the main randomized controlled trial are quasi-random, within each township (e.g. Pakokku).

### Performance and effectiveness

Table 2 presents our main results on program performance. Overall, program performance was high in both government-led and NGO-led villages. Only 2% of ineligible women were mistakenly enrolled in the program, although a much larger fraction (24%) of eligible women failed to enrol. Among those enrolled, 88% report receiving the full benefits for which they were eligible by the end of the program.

**Table 2.** The effects of delivering maternal cash transfers on program performance

	(1)	(2)	(3)	(4)	(5)	(6)
	Woman included, but ineligible	Woman excluded but eligible	Woman received transfer	Tot transfers	Woman received full benefits (frequency and amount)	Stay longer (>25 months)
GOV	−0.003 (0.007)	0.043 (0.052)	−0.013 (0.019)	1.823*(0.852)	0.197** (0.087)	−0.146*** (0.042)
Wild *P*-value	0.723	0.594	0.605	0.134	0.123	0.000
Observations	5110	857	5967	736	736	736
Mean NGO	0.02	0.24	0.12	20.07	0.78	0.15
Clusters	13	13	13	13	13	13

Notes: The table presents OLS estimates of the effects of delivering the maternal cash transfer program through government health workers (GOV) compared to an NGO on measures of performance. The sample includes the matched villages: 22 from GOV and 22 from NGO. The outcomes include: inclusion error, defined as whether an ineligible woman was enrolled in the program (sample includes all ineligible women) (1); exclusion error, defined as whether an eligible woman was excluded from the program (sample includes all eligible women) (2); whether an enrolled woman received at least one monthly cash transfer (sample includes all enrolled women) (3); total number of monthly cash transfers received since enrollment (4); whether an enrolled woman received the full amount of cash transfers since enrollment (5); whether an enrolled woman remained in the program beyond eligibility (6). Controls include (i) individual demographic controls, mother’s age and education, and head of household’s age and education for child-level analysis; mother’s age and education, and head of household’s age and education for mother-level analysis; (ii) village-level controls, including distance to large and small markets, main source of livelihood (agriculture, livestock, or casual labor), availability of government provided electricity, and participation in a concurrent WASH intervention. Standard errors are clustered at the cluster level. Wild bootstrapped (9999 reps) *P*- values are presented to take into account the small number of clusters. ***P <0.01, **P <0.05, *P <0.1.

Results in the first three columns reveal that the model of delivery has neither effect on the number of beneficiaries enrolled or reached, nor on their adherence to rules of eligibility. Women in government and NGO villages had the same probability of inclusion (column 1) and exclusion error (column 2), and a similar probability of receiving at least one cash transfer during enrolment (column 3). However, among those enrolled in the program (*N* = 736), women in government villages received a higher rate of benefits. Compared to beneficiaries living in NGO villages, those in government villages received 1.8 additional monthly cash transfers during enrolment (column 4) even though the effect is only statistically significant at the 10% level. They were 19.7% more likely to receive the full number and amount of transfers (column 5) and also 14.6% less likely to stay in the program beyond eligibility (column 6). However, the results on performance on the full sample (columns 4–6) are also only statistically significant at the 10% level when adjusting for the small number of clusters using wild bootstrapping methods (Supplementary Appendix Table S2). In the matched sample, only the coefficient on whether the beneficiary remains in the program beyond eligibility is statistically significant with wild bootstrapping.

Table 3 explores the impact of delivery model on child stunting, with the important caveat that this outcome was not among our prespecified primary outcomes of interest given that it was determined unlikely that we would have sufficient statistical power to detect an effect on stunting. The estimates reveal that the proportion of children stunted is 8.0 percentage points lower in government compared to NGO villages (column 1), and that this appears to be driven by changes (6.8 percentage points) in moderate stunting. This is also reflected in the HAZ score, which is 0.33 standard deviations higher (column 4). When estimates consider the low number of clusters, *P*-values confirm that the change in HAZ score remains statistically significant at the 5% level. The results are robust to using the full sample of villages (Supplementary Appendix Table S3). Supplementary Appendix Figure S2 also confirms a right shift of the HAZ distribution in the government villages compared to the NGO, and a Kolmogorov–Smirnov non-parametric test for equality of distributions indicates a statistically significant difference in the distribution among the two groups (*P*-value = 0.003).

**Table 3.** The effects of delivering maternal cash transfers on child stuntin

	(1)	(2)	(3)	(4)
	Prop. of children stunted	Prop. of children moderately stunted	Prop. of children severely stunted	HAZ score (WHO)
GOV	0.080* (0.037)	−0.068* (0.036)	−0.012 (0.009)	0.330*** (0.079)
Wild *P*-value	0.157	0.183	0.264	0.033
Observations	626	626	626	626
Mean NGO	0.30	0.24	0.05	−1.38
Clusters	13	13	13	13

Notes: The table presents Ordinary Least Squares estimates of the effects of delivering the maternal cashtransfer program through government health workers (GOV) compared to an NGO on measures ofperformance. The sample includes the matched villages: 22 from GOV and 22 from NGO. Outcomes includethe proportion of children stunted as children with Height for Age Z score (HAZ) < -2 (1); the proportion ofchildren moderately stunted as children with HAZ < -2 and >= -3 (2); the proportion of children severelystunted as children with HAZ < -3 (3); and, HAZ (4). Controls include (i) individual demographic controls,including child’s sex and age, mother’s age and education, and head of household’s age and education; (ii)village-level controls, including distance to large and small markets, main source of livelihood (agriculture,livestock, or casual labor), availability of government provided electricity, and participation in a concurrentWASH intervention. Standard errors are clustered at the cluster level. Wild bootstrapped (9999 reps) *P*-values are presented to take into account the small number of clusters. ***P <0.01, **P <0.05, *P <0.1.

### Other outcomes

We also investigate the extent to which positive impacts on malnutrition are associated with changes in maternal health behaviours (Table 4). Across interventions, we find similar total food consumption, hand washing practices and proportion of mothers having at least four antenatal care visits as recommended by WHO (columns 1–3). We also find similar proportions of children ever breastfed (99%) and that received colostrum (95%) (columns 4–5). In addition, we explore maternal health knowledge and confirm that mothers’ level of knowledge is high and comparable across government and NGO villages (Supplementary Appendix Table S4), as is self-reported cash usage by the respondents (Supplementary Appendix Table S5).

**Table 4.** The effects of delivering maternal cash transfer on women and children’s behavior

		(1)	(2)	(3)	(4)	(5)	(6)
		Mother			Child		
		Tot. Food consumption	Index of hand-washing behaviour	Prop. of mothers with at least 4 ANC visits	Prop. of children ever breastfed	Prop. of children received colostrum	Any illness (2 weeks)
	GOV	378.589 (967.708)	0.173 (0.339)	−0.063 (0.044)	0.004 (0.004)	0.012 (0.011)	−0.051** (0.022)
	Wild *P*-value	0.740	0.697	0.319	0.609	0.479	0.106
	Observations	661	661	574	630	630	630
	Mean NGO	22 696.60	3.07	0.86	0.99	0.95	0.97
	Clusters	13	13	13	13	13	13

Notes: The table presents Ordinary Least Squares estimates of the effects of delivering the maternal cash transfer program through government health workers (GOV) compared to an NGO on mothers and children’s behavior. The sample includes the matched villages: 22 from GOV and 22 from NGO. Outcomes include a numerical index (1 to 9) of hand washing practices combining whether mothers report always washing hands after cleaning a baby’s bottom, after using the toilet, before preparing and eating food, before feeding children, after disposing of baby feces, before and after handling children, and on other occasions (1); the proportion of mothers receiving at least 4 Antenatal Care visits with skilled health personnel, as defined by WHO standards (2); total food consumption, winsorized at the 99th percentile level (in last 7 days, in MMK, 3); the proportion of children ever breastfed (4); the proportion of children who received colostrum (4); the proportion of children with any illness in the past two weeks (6). Controls include (i) individual demographic controls, including child’s sex and age, mother’s age and education, and head of household’s age and education for child-level analysis; mother’s age and education, and head of household’s age and education for mother-level analysis; (ii) village-level controls, including distance to large and small markets, main source of livelihood (agriculture, livestock, or casual labor), availability of government provided electricity, and participation in a concurrent WASH intervention. Standard errors are clustered at the cluster level. Wild bootstrapped (9999 reps) *P*-values are presented to take into account the small number of clusters. ***P <0.01, **P <0.05, *P <0.1.

However, children in government villages are 5.1 percentage points less likely to have had any illness in the previous 2 weeks (including fever, diarrhoea and pneumonia), and this result remains close to statistically significant at 10% level when taking into account the small number of clusters (wild *P*-value = 0.106).

### Key factors of success (qualitative evidence)

To better understand the mechanisms underlying the results of the study and identify the key factors that favour government delivery in this setting, we conducted focus group discussions with the 11 health workers in government villages on their experience with the program. There are several reasons why government health workers may have performed better in the delivery of the maternal cash transfer program, which materialized in health benefits for the young children.

First, the agents involved in each mode of delivery were different to start with, based on their current profession. While agents across treatment arms were mainly women, government health workers were already a ‘trusted presence’ in the community through their sub-rural health centres, and they were a regular point of contact for women through the provision of basic health services such as antenatal care. The program may have been enhanced by the complementarity between the expertise of government health workers in health services provision for pregnant women and young children, and the field visits required for cash delivery. In contrast, loan agents were largely considered outsiders of the communities, coming in only for their business as part of their micro-finance institution.

In terms of ‘performance incentives’, it is possible that the civil service model in Myanmar is more of a performance-based meritocracy than what is found in the NGO sector (Hongbin and Zhou, 2005; Serrato *et al*., 2019). Myanmar introduced performance-based evaluations for civil servants who report to the union government and ministries (Nixon *et al*., 2013). Our respondents describe that, while their post at a sub-rural health centre is assigned by union-level Ministry of Health and Sports’ administrators, the final decision on filling vacancies is made by state and regional health departments. Despite a lack of transparency in the assignment process, they report that they are allowed to specify preferences over posts, that everyone who applies receives an assignment, and they do not face any threat of removal. However, perception survey data on ethics in the Myanmar Civil Service indicate that civil servants are highly sceptical about how much promotions and postings are decided based on merit (UNDP, 2016). Thus, while this channel is possible, it seems less likely to be a key difference in performance incentives between government health workers and loan agents in this setting.

However, although government health workers did not reported concern over losing their posts, our focus group discussions reveal that they are nonetheless subject to monitoring by supervisors at the union, state and township health departments, which can affect their career advancement. For instance, midwives found to be underperforming must undergo further training by higher-level health officials. According to the respondents, government health workers are also expected to report and explain in detail cases of maternal and child mortality in their communities, which may be the key detail explaining their added value for maternal cash transfer delivery. In particular, the program encouraged pregnant women to report their pregnancies and regularly attend antenatal visits. In this sense, the program also benefited government health workers by enabling them to identify high-risk pregnancies or sick children to provide care more quickly. In line with our main findings on child health, they also state that they readily provide consultations and referrals to hospitals when they identified sick children during field visits.

As far as ‘motivation’ is concerned, it is also possible that government health workers outperformed loan agents operating in neighbouring villages because the cash transfers provided a new opportunity for them to gain trust and reputation in the community, which could potentially be leveraged to recruit clients for their private practice. Government health workers report that, in addition to their regular out-patient services at the sub-rural health centre at specific hours during the day, they also spend time during off-hours providing private health services in communities, as has been documented for public sector workers in health and education sectors in other settings (Jayachandran, 2014). For example, respondents report that, when the Ministry of Health and Sports does not provide them with enough drug supply, they often purchase drugs for the community and charge patients a fee. They also describe providing medical consultations in addition to the services they are expected to deliver as part of their post. In addition, although they do not request fees for regular services, such as delivery, they mention that households regularly provide them with cash or in-kind gifts. Overall, according to respondents, their increased community engagement as part of maternal cash transfer delivery did not influence their public or private health care practices, but there is no way to rule out the possibility that they were more motivated to regularly deliver program benefits because of corresponding gains to their reputation as health providers in these communities.

A final possibility is that government health workers had more intrinsic motivation to improve community nutrition in program villages than did NGO loan agents, by virtue of working in health care rather than the microfinance sector. In national survey data, civil service personnel in Myanmar report high levels of motivation such as being proud to belong to the civil service (UNDP, 2016). Likewise, our own qualitative data indicate that government health workers in our setting are highly motivated to improve the health of mothers and children in their communities. On the other hand, we know little about levels of intrinsic motivation in the NGO microfinance sector in Myanmar, although and—as discussed previously—it is generally assumed that international NGO workers have higher levels of intrinsic motivation than public sector workers in settings with weak government institutions (Ghatak, 2005; Werker and Ahmed, 2008).

## Discussion

Our study contributes novel evidence to the current literature in health service delivery (Khan and Ahmed, 2002; Banerjee *et al*., 2008; Reinikka and Svensson, 2010) by showing that social welfare programs aimed at improving health can be more effectively delivered through government actors than through loan agents of an NGO in the setting of rural Myanmar. Overall, there is no strong indication that the delivery mode, or resulting difference in levels of or consistency of benefits received, influenced maternal health behaviours predicted to influence child stunting. However, we do see evidence that the delivery mode influenced child exposure to illness.

Focus group discussions, we conducted with government health workers in program villages suggest that this pattern may be driven by greater attention to child health from qualified program staff. Specifically, government health workers report that the maternal cash transfer program helped them to identify pregnant women and children at high risk of health complications. Hence, we speculate that the monthly delivery of cash transfers by government health workers allowed beneficiaries to connect more frequently with qualified health personnel, who provided advice and referrals when encountering sick children. This channel may also be responsible for the significant difference in rates of stunting across delivery models, as illness episodes are a potential source of chronic malnourishment in children (Dewey and Mayers, 2011).

In light of this qualitative evidence, we believe that a likely channel through which government health workers were better able than loan agents to promote child health in program villages is the complementarity between their expertise in the provision of community health services—which entails a reliable presence in the community and regular interactions with targeted beneficiates—and the field visits required for cash delivery. In addition, monitoring from higher-ranked officials and their overarching performance incentives for maternal and child health objectives was another likely factor of success. However, we cannot fully rule out that private incentives or intrinsic motivation contributed to the difference between the performance of government health workers and loan agents.

This study is not without limitations. First, while our large experiment had the statistical power to detect the primary outcome of stunting (Field and Maffioli, 2024), this secondary study primarily had statistical power to detect measures of performance. Second, we lack data on micro-costs of the program for each mode of delivery to draw conclusion on the comparative costs and thus the cost-effectiveness, and we hope further research could shed additional light on the cost dimension. Despite these caveats, this study adds insights on the key factors of success in delivering maternal cash transfers in a lower income country, which is a novel and policy relevant setting to explore.

## Conclusion

This study investigated the relative effectiveness of different mode of delivery of maternal cash transfers in rural Myanmar and identified key factors of success. Using a variety of data sources, we measured program delivery performance across villages assigned in an arguably quasi-random fashion to deliver the program through either government health workers or loan agents of an NGO. We also measured differences across delivery models in terms of the program effect on maternal health behaviours and child illness and stunting. We find evidence that the delivery of cash transfers through government health workers outperformed delivery through loan agents. Ultimately, beneficiaries whose monthly cash transfers were delivered by government health workers received greater levels of and consistency of cash transfers, and experienced greater improvements in child health.

Our results shed light on the potential benefits of utilizing government health workers for the delivery of cash transfers in Myanmar and showcase the potential for positive spillovers of program staff interactions onto child health when staff are trained health professionals. These findings are immediately relevant to future efforts to scale up the maternal cash transfer program throughout the country, where the maternal cash transfer program was projected to reach 2.25 million beneficiaries and 0.32% of GDP by 2024 (The Republic of the Union of Myanmar, 2014).^6^ More generally, these findings suggest that maternal cash transfer programs should be delivered by those actors who best fulfil the critical success factors identified in this study. While, in Myanmar, government health workers were the best positioned to successfully deliver the program due to their trusted presence and past interactions with targeted beneficiaries, complementarities between their expertise and the program, as well as performance incentives based on specific health objectives along with top-down monitoring, this might not be the case in other countries. As cash transfers gain traction in many other low- and middle-income countries as a social protection mechanism (Gentilini, 2022), these findings have increasing policy relevance. Decision makers considering maternal cash transfer programs in other settings should ensure that these factors of success are present as much as possible in the actors chosen for delivery.

## Supplementary Material

czae048_Supp

## Data Availability

Data available on request.

## Footnotes

1. A large literature points to a theoretical and empirical relationship between state capacity and the provision of public services (Besley and Ghatak, 2001; Acemoglu, 2005; Besley and Persson, 2009).

2. Sustainability is also a potential way in which the quality of care provided by government and NGO workers differs, although this is something that is only evaluable with a long-term study. For example, there is evidence that NGOs might be less likely to maintain a permanent presence, especially in rural communities that are more costly to serve and where organizations can reach less people than in urban areas (Sharma and Zeller, 1999; Barr and Fafchamps, 2006).

3. The pilot program was supported by Save the Children International.

4. The trial was registered on AEA RCT registry. Registration number: AEARCTR-0004189. https://www.socialscienceregistry.org/trials/4189.

5. Although we did not register a Pre-Analysis Plan for this secondary study, power calculations are detailed in the 2016 Research Protocol that was delivered to our government partner for study approval prior to the onset of the RCT (https://elisamaffioli.files.wordpress.com/2021/12/160404_legacy_rct_research-protocol_final.pdf). At that point in time, the details of this secondary study were not fully defined, and we assumed we would be able to detect a difference between two different models, but, as specified, the comparison could have been between GOV model and MFI (=NGO) model or mobile money and MFI (=NGO) model. We assumed power at 0.8, alpha at 0.05 to detect a 10-percentage point difference in the proportion of beneficiaries that do receive cash transfer (or that do receive cash transfer in time or the correct amount, from a baseline of 70%). Given these assumptions, calculations required a minimum of 313 participants in each treatment arm, for a total of about 626 in the two intervention areas.

6. These projections are as of February 2021. The program was abruptly stalled after the military coup of February 2021. Current efforts to deliver cash transfers are led by international or local organizations.

## Reflexivity statement

The authors include two females, one senior and one junior faculty, and one male from Myanmar. The authors specialize in economics, nutrition and global health policy. The female authors have extensive experience conducting RCTs and quantitative analysis in the Global South, including South East Asia. The male author has extensive experience working in nutrition in Myanmar, in close collaborations with government and non-government organizations. Nicholus Tint Zaw who is Burmese is currently at IFPRI, but he was based in Myanmar, working for Save the Children and then for Innovations for Poverty Action during the study period.
